# Individual and relational dynamics perceived to influence the sexual behaviour of adolescents in Ethiopia: a qualitative study

**DOI:** 10.3389/frph.2024.1348953

**Published:** 2024-08-06

**Authors:** Semere Gebremariam Baraki, Gloria Thupayagale-tshweneagae

**Affiliations:** ^1^ Department of Public Health, Menelik II Medical and Health Science College, Kotebe University of Education, Addis Ababa, Ethiopia; ^2^ Health Studies, University of South Africa, Pretoria, South Africa

**Keywords:** adolescents, exploratory, individual level, influence, relational level, sexual behaviours

## Abstract

**Background:**

There are 1.2 billion adolescents in the world today, more than ever before, making up 16% of the world's population and nearly one-fourth of the total population in Sub-Saharan Africa. Adolescents are facing life-threatening health challenges attributed to sexual and reproductive health issues such as unwanted pregnancies, unsafe abortions, and sexually transmitted infections, including the human immunodeficiency virus, and acquired immunodeficiency syndrome. The aim of this research is to explore the individual and relational levels of factors that drive adolescents to engage in risky sexual behaviour.

**Methods:**

A qualitative phenomenological study design was used from February to June 2020. Adolescents and health professionals were selected purposefully. A total of 12 individual in-depth interviews, five focus group discussions with adolescents, and eight key informant interviews with health professionals were conducted using a semi-structured guide. Data analysis was performed using thematic analysis with ATLAS Ti version 7 software. Credibility, dependability, transferability, and confirmability were used to ensure the trustworthiness of the data.

**Results:**

In this study, two themes were identified; individual level factors such as sexual desire and emotion driven sex, limited knowledge of sexual and reproductive health, and a permissive attitude towards sexual activities drive adolescents to engage in risky sexual behaviour; and relational level factors such as, limited family support and involvement, negative peer pressure and influence, male partner dominance during the partnership, and pressuring females to engage in sexual intercourse were perceived factors influencing adolescents to engage in risky sexual behaviour.

**Conclusion:**

Various individual-level and relational-level factors are influencing adolescents to engage in risky sexual behaviour. Socially and culturally acceptable, comprehensive sexual education should be provided for in-school and out-school adolescents to enhance their knowledge, attitude, and skill about sexual and reproductive health. Interventions at the peer and partner level should be considered to enhance the life skills that enable them to resist pressure from peers and their partners. Child-parent communication on sexual and reproductive health matters should be promoted.

## 1 Introduction

Globally, adolescents aged between 10 and 19 account for 1.2 billion (16%) of the world population and nearly one-fourth of the total population in Sub-Saharan Africa (1). In Ethiopia, one-third of the total population were adolescents and youth aged between 10 and 24 years old; approximately half of those were girls (2). Adolescence is the period between childhood and adulthood, and it is an experimental age in which adolescents become vulnerable to different risks that may result in various physical and psychological health problems (3). Adolescents' experiences include physical, hormonal, and cognitive changes (4). Emotionally, they develop a sense of identity during late adolescence; social involvement, peer interaction, and sexual interest, develop in this phase. Different behavioural experimentation is seen in early adolescence, risk taking in middle adolescence, and later adolescents learn to assess their own risk taking (5). Adolescence is the time to explore and understand sexuality. Sexual curiosity in adolescence led to exposure to pornography, indulgence in sexual activities, and increased the vulnerability to sexual abuse (6). Adolescents are somewhat at an increased level of vulnerability for different health problems, including sexually transmitted diseases, when compared to the adult population (7). Risky sexual behaviour is characterized by different dangerous behaviours such as premarital sex, multiple sexual partners, and unprotected sex. Such dangerous sexual behaviours are reported to end up with unpleasant health outcomes from sexually transmitted infections, including, HIV/AIDS, unwanted pregnancies, and unsafe abortions (8, 9).

Worldwide, 2.1 million adolescents aged 10–19 years were living with HIV (Human Immunodeficiency Virus) and 55,000 Acquired Immune Deficiency Syndrome (AIDS) deaths among adolescents in 2016, which is mostly due to risky sexual behaviour (10). Sexually active adolescents aged 15–19 years are at greater risk of acquiring sexually transmitted diseases compared with older adults (11).

Adolescent sexual and reproductive health remains a major public health issue in sub-Saharan Africa, especially for adolescent girls (12). Adolescent girls and young women (AGYW) aged 15–24 years have the highest risk for HIV and STIs across sub-Saharan Africa (13). Similarly, young people in Ethiopia are at risk of various SRH problems, such as sexually transmitted infection, including HIV, unwanted pregnancies and unsafe abortions, and various pregnancy related complications (14). For instance, the pooled estimated prevalence of teenage pregnancy in Ethiopia was 23.59 (15); 44% of pregnancies among adolescents were unintended, of which 46% ended in abortion (16). HIV prevalence among adults with a sexual debut before the age of 15 years (6.8%) was higher than that among those with a sexual debut between ages 20 and 24 years (2.7%). The prevalence of HIV infection was higher for women whose sexual debut was between ages 15 and 19 years or ages 20 and 24 years than for men with the same age of sexual debut (17). According to EDHS, the highest prevalence of HIV (4.8%) was observed in the Gambela regional state, followed by Addis Ababa (18). Evidence in Ethiopia indicates that the prevalence of risky sexual behaviour among adolescents attending school ranges from 13% in Humera, western Tigray (19), to 71.2% in Addis Ababa (20).

Various factors, such as personal, family, peer, school, and community groups, were found to be contributing factors to high-risk sexual behaviour among adolescents (21). In addition to this, individual lifestyle, and structural factors play an important role in influencing sexual behaviour among university students (22). Poor knowledge of sexual and reproductive health is another factor that influences adolescents' health-seeking behaviour (23). Some adolescents were not armed with the required information on sexually transmitted infections (24). Based on the study in Ethiopia, poor social support, living out of the family, experiencing parental neglect, and drinking alcohol were statistically significant risky sexual behaviours (25). Substance abuse, watching pornography, and nightclub visits were associated with risky sexual behaviours among secondary and above educational level students in Ethiopia (26).

Adolescents and youth health (AYH) programs, including those focused on SRH and youth development, have gained traction in Ethiopia to satisfy the requirements of this age group; however, teenagers and young people in Ethiopia still face high rates of morbidity and death due to a variety of issues, including teenage pregnancy, unintended pregnancies, poor nutrition, HIV and STIs, unsafe abortion, early and child marriage, and unmet family planning requirements (27).

Though quantitative study findings done in Addis Ababa indicated that, 20.4%, 70%, and 40.3% of youth and adolescents were engaging in risky in 2015 (28), 2017 (29), and in 2018 (30) respectively, there is no qualitative studies that explore and explain why and how adolescents are engaging in risky sexual behaviour in Addis Ababa. So, the aim of this study is to explore the individual and relational level factors that influence adolescents to engage in risky sexual behaviour.

## 2 Methods

Reporting adheres to the Consolidated Criteria for Reporting Qualitative Research (COREQ).

### 2.1 Study setting and period

The study was conducted in urban setting in Addis Ababa, the capital city of Ethiopia from February to June 2020. In Addis Ababa, there are 103 public health centers, 11 public hospitals, 33 private hospitals, and 270 pharmacies (31, 32), and there are a total of 106 functional youth centres (33). Study participants were selected from health centres, hospital, youth centres, and nongovernmental organization namely the Family Guidance Association of Ethiopia (FGAE) in Addis Ababa. FGAE has a mission to deliver comprehensive, integrated, quality, and gender sensitive SRH programs and services focusing on youth and underserved, vulnerable populations (34).

### 2.2 Study design

A qualitative Phenomenological study design was conducted using in-depth interviews (IDIs), focus group discussions (FGDs), and key informant interviews (KIIs) data collection techniques to achieve the objectives of the study. This design enables the investigator to explore the phenomena from the perspective of the participant being studied (35). We approached the research questions from the perspectives of health care provider and adolescents about sexual behaviours of adolescents based on individual level, and relational factors.

### 2.3 Population and sampling technique

The data was collected from 51 individuals (8 health professionals and 43 adolescents). The characteristics of qualitative sampling and sample size are determined by the study designs and conceptual requirement than representativeness (36). In qualitative research, participants are selected due to lived experience with the area of interest, purposeful sampling, to get rich and thick data about the phenomenon of interest and data collection continued until data saturation had been reached or no new concept is emerging (37). The researchers used purposeful sampling techniques till saturation had been reached. In a largely deductive approach, saturation may refer to the extent to which predetermined codes or themes are adequately represented in the data whereas inductive approach thematic saturation in related to the non-emergence of new codes or theme (38). So, the sample size was determined by information redundancy or saturation level, which occurred when no new information, theme, or coding emerged from the data. To ensure saturation, the data was reviewed at the end of each interview day for the presence of codes or categories, as well as the necessity for further interviews in a preliminary manner. Furthermore, the researcher ensured that interviews were no longer generating new information since newly conducted interviews tend to be redundant with previously collected data. After several similar responses or ideas, the recorded data no longer generates fresh code. Data saturation was assured after 12 IDIs with adolescents, 8 KIIS with health professionals, and 5 FGDs with adolescents.

The researchers used purposive sampling technique based on eligibility criteria listed below. Adolescents who fulfilled the following criteria were included in the study: Adolescents who receive SRH services in the selected health institution or receive SRH services and other library, and/or recreational services in youth during the data collection period, Adolescents with written consent to participate in the study, Adolescents between the ages of 15 and 19. Health professionals who fulfilled the following criteria were included in the study; age 18 years and above, responsible for any sexual and reproductive health service for adolescents in the selected health institutions and youth centers, working as SRH service provider in the selected health institutions or youth centers for at least 1 year, provided written consent to participate in the study.

### 2.4 Data collection tool and procedure

A semi-structured interview guide was prepared after reviewing relevant literature (39, 40) and different probing questions were added before and during the data collection process. SGB and GBT carefully crafted the interview guides in English and SGB translated to Amharic languages. The interviewed guid was pretested before actual data collection by two adolescents and two health professionals who were not included in this study. Based on the inputs from the pretest, the interview guide was revised for actual data collection. The principal investigator Semere Gebremariam Baraki (SGB) worked with the Addis Ababa regional health bureau, adolescents and youth case team leader, and the head of selected health care organizations and youth centres. First the principal investigator, in collaboration with head of selected health care organizations and youth centers, purposefully selected various service areas such as voluntary counselling and testing (VCT), Abortion service units, family planning service unit, antiretroviral therapy (ART) clinics, adolescents, and youth outpatient departments. Health professionals working in the selected department and were requested to identify adolescents who met the inclusion criteria. The principal investigator informs adolescents about the purpose, objective, risk, and benefits of the research. The researcher provided “yes” or “no” question on previous history of sexual intercourse to fill individually. Adolescent who said “yes” for previous history of sexual intercourse asked their willingness for participating in the research process in the IDIs.

The principal investigator and the head of youth center selected different service areas, recreation area, clubs, and libraries in the selected youth center and communicated with health professionals to call adolescents for orientation about the research. After giving briefing orientation, the researcher himself identified those who fulfilled the criteria purposefully for the FGDs. In both FGDs and IDIs, assent form was given to adolescents who were willing to participate in the study and Consent form was given to each adolescent for his/her parents to review and determine whether to allow their children to participate in the study the adolescents were found less than 18 years old. Written informed consent was obtain from adolescents' parents/cares before the data collection started. The data collection was conducted immediately after the biographic data filled if the adolescents were found above 18 years old. But the researcher appointed the participants for the convenient time and place for the next time for adolescents who were less than 18 years to secure parents'/care givers' written informed consent.

For the sake of KIIs, the principal investigator met the head of health institution and asked to recommend senior health professionals who have experience with adolescents sexual and reproductive health. He explained the purpose and procedure of study and asked them for voluntary participation. Consent form was secured from health professionals before conducting in-depth interviews. Data collection was carried out by the principal investigator (SGB) and two experienced female research assistants. The two female research assistants were assigned to take notes during the FGDs after a brief training about the research ethics and process by SGB. The research assistants were fluent in local Amharic language (FGD participants' working language), one has MSc degree and the other has PhD in public health, and both had experience in qualitative research. All the data were collected using face-to-face interviews.

The biographic data was filled in separately; any personal identifiers were not recorded during the interview process. Participants were given all the relevant information relating to the study, which includes the title, purpose of the study, benefits, and any potential risks. Participants have had the opportunity to ask questions freely and without fear. IDIs, FGDs and KII were all audio-recorded after obtaining participants' permission to record. Data collection took place at different locations depending on participants. The FGDs were conducted in youth centers, IDIs were collected in the youth centers and Health care organization, while the KIIs were conducted in their offices, and the clinics. The data collection process lasted on average 40–75, 30–75, and 75–115 min for IDIs, KIIs, and FGDs, respectively.

### 2.5 Data processing and analysis

All the audio recorded interviews were transcribed verbatim to Amharic by principal investigator immediately after the interview. The principal investigator anonymized the names of the participants during transcriptions; the names of the participants in the FGDs were substituted as participant one (P1), Participant two (P2), and participant three (P3) and so on. The names of adolescents participated in IDIs were also replaced by adolescent one (A1), adolescent two (A2), and adolescent three (A3) etc. Similarity the names of health professionals who involved in the KIIs were anonymized as health professional one (HP1), health professional two (HP2), and health professional three (HP3) and so one. The transcribed data were translated into English. The verbatim transcription and careful translation were used to guarantee the accuracy of the original messages of the interview. In this case the recordings were listened to several times to form a general structure. All 25 transcripts were analysed with ATLAS.ti version 7 software. Thematic analysis was used to identify emerging themes using a six-step approaches: familiarizing with the data, generating initial codes, searching for themes, reviewing themes, defining, and naming themes, and writing the report (41). Thematic analysis was the most ideal approach to capture all aspects of data deductively and inductively. SGB coded the data and GBT comment and approve the coding. After reviewing four transcripts and field notes, he developed a code tree with a list of deductive and inductive codes from the data. The deductive codes came from topics in the interview guide and inductive codes captured new themes that emerged in the data (42). SGB developed a codebook, and this codebook was reviewed by the research supervisor GBT, and revisions were made where necessary till the end of data analysis. Identified discrepancies were discussed, resolved, and reviewed further until consensus was reached. The researchers systematically grouped the sub-categories into categories, and categories into general themes. We organized the findings by major themes, and we discussed minor themes in the manuscript and situated them within the broader literature. Participants' quotations were presented to illustrate themes and findings.

### 2.6 Trustworthiness

In this study, the constructs of credibility, transferability, dependability, and confirmability were enhanced (43, 44). Prolonged engagement and persistent observation; data, space, time, person, method triangulation; peer debriefing is among of techniques to maintain credibility. So, the principal investigator spent a lot of time in the field with study participants in maintain prolonged relationship and building good rapport, and gain deep understandings of the participants' experiences, and context to earn their trust and familiarize us with them and the data collection site to collect thick and credible data. The supervisor reviewed and approved the preliminary results, or subcategories, categories, and themes. The supervisor's remarks indicate that corrections were made. The data was collected from different sources; hospital, youth centers, and health centers and data were collected by individual interview, focused group discussion. Data was collected from health professionals and adolescents well involved as study participants for the purpose of triangulation techniques we employed.

To maintain dependability, four Study participants (two health professionals and two adolescents) were given raw transcripts by the researchers and inquired whether the transcripts accurately reflected the conversations that took place during the data collection phase. The findings of this study were audited and verified by advisors so that an outside individual can examine the data. Each process was documented, and audio records were used for cross-checking.

To maintain conformability, all data collected from the fieldwork were kept and were frequently checked and rechecked, and agreed upon between the investigators throughout data collection, analysis, and interpretation phase. The interview guide was translated into local languages and the inquiry was conducted in the language of the participant's choice to reduce bias and error.

To maintain transferability, The researchers provide a thorough explanation of the methodology they employed, including the steps involved and duration of the data collection, analysis, and presentation processes. All the audio recordings, transcripts, subcategories, categories, and themes were preserved by the researchers.

### 2.7 Ethical considerations

Ethical approval was obtained from the University of South Africa, Department of Health Studies, higher degrees committee with the reference number Rec-012714- 039 (NHERC) and from Addis Ababa city administration Public Health Research and Emergency Management Core Process (AACAPHREM) with the reference number of አ/አ/ጤ/30/703/227. Permission was sought from the heads of health care organizations and youth centers. Written informed consent was obtained from each participant and/or minors' legal guardian or next of kin before the interview began. The researcher explained that participation was voluntary and that the participants could withdraw from the research at any given time without any repercussions. Minors were only allowed to give assent and participate in the study after written informed parental consent. Participants were assured of strict confidentiality regarding the information collected; that only aggregated data will be made available as part of scientific and public dissemination. Participants were given all the relevant information relating to the study that includes the title, purpose of the study, benefits, and any potential risks. Compensation of $5 was given to all participants for their time. Participants had the opportunity to ask questions freely without any fear. The data were collected from anonymized responses; security techniques were maintained starting from data collection, analysis, and presentation. All the data were protected using a password, and the audio was immediately copied to the computer and saved using a password and deleted from the tape recorder.

## 3 Results

### 3.1 Participants

The researcher collected data from adolescents, and health professionals; 12 IDIs, 8 KIIs, and five FGDs. Out of the five FGDs, three were conducted with male participants, while two were conducted with female participants. Sociodemographic characteristics such as age, sex, religion, grade, living condition, history of sexual exposure, living situation, and the sub-city were collected during the data collection. The adolescents were represented as P1, P2, P3, etc. Codes during the interview. KII represents the key informant interview, IDI represents the individual in-depth interview, and FGD represents focus group discussion. A total of 43 adolescents participated in the study; out of this, 30 were school attendants, and 13 were out-of-school adolescents (Table 1).

**Table 1:** Socio-demographic characteristics of adolescents who participated in individual. In-depth interviews and focus group discussions.

Variable	Category	Frequency	Percentage
Sex	Male	25	58.1
	Female	18	41.9
Data collection type	IDIs participants	12	27.9%
	FGDs participants	31	72.1%
School attendance	In school	30	69.8%
	Drop out of school	13	30.2%
Reason for last visit of health institution/youth center	Voluntary counselling and testing	7	16.3
	Condom uptake	4	9.3
	Sexually transmitted infections	1	2.3
	Recreation	10	23.3
	Library use	8	18.6
	Medical service	6	14.0
	Training	6	14.0
	ART service	1	2.3
Total		43	100%

The socio-demographic characteristics of health professionals who participated in the study were also presented. The average age of the study participants in this group was 35.75 years. The oldest was 54 years old, and the youngest was 24 years old. They had an average of 13.50 years of work experience, with a maximum of 32 and a minimum of 4 years of work experience. There were 4 females and 4 males. Regarding their profession, four of the totals were clinical nurses, two were nurse counsellors, and the rest were health officers. All participants were working in a health canter, hospital, NGO, or youth centre (Table 2).

**Table 2:** Socio-demographic characteristics of health professionals participated in KIIs.

Variable	Category	Frequency	Percentage
Sex	Male	4	50%
	Female	4	50%
Profession	Clinical nurse	4	50%
	Nurse counsellor	2	25%
	Health officer	2	25%
Educational background	Diploma	3	37.5%
	Degree	5	62.5%
Marital statues	Single	4	50%
	Married	3	40%
	Divorced	1	10%
Total		8	100%

### 3.2 Qualitative findings

In this research two themes, 7 categories, and 23 subcategories were identified. Theme one is individual-level factors, and theme two is relational-level factors. The themes, categories, and subcategories are briefly summarized in (Table 3).

**Table 3:** Themes, categories, and sub-categories of the analysis.

Themes	Categories	Sub-categories
1. Individual-level Influence	1.1 Biological factors	1.1.1 Having sexual desire
		1.1.2 Emotion driven
2. Relational level influence	1.2 A gap in knowledge and skill of SRH	1.2.1 Limited knowledge about STIs/HIV
		1.2.2 Stereotyping and misjudgement
		1.2.3 Limited knowledge and skill of condom use
	1.3 Attitude on sexual behaviour	1.3.1 Attitude on early sexual initiation
		1.3.2 Attitude on condom use
		1.3.3 Attitude toward multiple sexual partners
	1.4 Behaviour stigmatization and judgemental	1.4.1 Unplanned sexual relationship
		1.4.2 Engaging in substance abuse
		1.4.3 Deceiving behaviour
		1.4.4 Dressing style
		1.4.5 Transactional sex
	2.1 Gap of family involvement in sexual and reproductive health behaviour modelling	2.1.1 Child parent communication about SRH
		2.1.2 Parental control and supervision
		2.1.3 Parenting style
		2.1.4 Child parent conflict
	2.2 Peer related factors	2.2.1 Negative peer norm
		2.2.2 Peer pressure/influence
		2.2.3 Peer to peer communication
	2.3 Partner and relationship related factors	2.3.1 Failing in relational problem
		2.3.2 Male dominance in deciding condom use
		2.3.3 Pressuring female to sexual activities
		2.3.4 Exposing to sexual violence

#### 3.2.1 Individual level influence

##### 3.2.1.1 Biological factors

Most study participants indicated that adolescents engaged in risky sexual behaviour because of Having sexual desire, and emotion-driven: adolescents fail to use condoms due to discomfort, need for more sexual satisfaction, forgetfulness during high sexual climax, and enjoying their sexual pleasure and without having the plan to have sexual intercourse. Besides, some study adolescents explained that sexual intercourse is a natural desire and difficult to control, and an individual cannot remain a virgin throughout their life; sexual intercourse might be ok if both sexual partners like to satisfy their sexual instincts. They explained that early sexual initiation can help them in their future relationships and develop experience.

“*The adolescents want to have free sex. They assume that sex using a condom means eating a banana with its rind.*” *(KII, 54 years old, male)*

“*Most of the time, adolescents have emotion-driven sex; therefore, they forget to use a condom. The other reason is that they don't have planned sex.*” *(KII, 54 years old, male)*

“*In my view, if a boy and a girl agree and if they have tested for HIV, there is no problem if they have it (sex). But before that, they must make sure that they are HIV negative.*” *(FGD, 19 years old, female)*

##### 3.2.1.2 A Gap in knowledge and skill on sexual and reproductive health

Participants revealed that adolescents have limited knowledge and skill in sexual and reproductive health. The narratives revealed that adolescents worry more about pregnancy than other sexually transmitted diseases, including HIV, because they lack knowledge of sexually transmitted diseases and have limited knowledge and skill in using condoms. In addition to those, misconceptions and myths about condom use were stated, and there were different types of stereotyping and miss-judgment-based appearances on condom use. Adolescents might not use condoms if their sexual partner looks healthy, does not have any symptoms of disease, is a virgin, is not a commercial sex worker, has decent behaviour and is physically attractive. However, such stereotyping and misjudgement-based physical appearance were explained by a few participants.

“*If I have sex without a condom suddenly, the first thing that comes to mind is pregnancy. If I have a baby now, what do I do since it was done without the permission of my family?*” *(IDI, 17 years old, female).*

“*When you see girls who have skin rashes on their faces, you may think that they are HIV positive. But with beautiful girls, a male can lose control, and he can have sex without it (condom).*” *(IDI, 18 years old, male).*

Knowledge and skill of using condoms vary among adolescents. Adolescents who have used a condom explained that they have the knowledge and skill to use condoms. However, there are also adolescents who lack the knowledge and the skill of using condoms. Adolescents explained their concerns about condom use: it can burst, get stuck in the womb, and cannot prevent STIs and HIV/AIDS 100%; they consider it only prevents 90%-95%; and it can cause infection in the sexual organs. Few of them perceive that it cannot prevent pregnancy and some communicable diseases, such as hepatitis.

“*Most of the time, the cause of unplanned pregnancy and diseases is not knowing how to use a condom.*” *(FGD, 19 years old, male)*.

“*I think it can prevent 90% of diseases or sexually transmitted diseases. I do not think a condom prevents unwanted pregnancy; it can protect me from sexually transmitted disease, but I do not think that it protects me from being pregnant*.” *(FGD, 17 years old, female)*

On the other hand, one female adolescent indicated that adolescents usually fear pregnancy more than HIV/AIDS because they do not have detailed knowledge about the seriousness of the disease. She added that she feared pregnancy and HIV/AIDS when she was younger and had little knowledge. But after she understood the disease, she better feared the disease than pregnancy. On the other hand, adolescents understand the risky severity of HIV as get older, as indicated by one senior health professional from the key informant.

“*It is terrifying. HIV/AIDS is very terrifying now. Pregnancy is not terrifying. I have never used a condom, but I think I should use it.*” *(IDI, 19 years old, female)*.

“*Let me tell you what I experienced recently. Two lovers (boy and girl) have been together in friendship for the last two years and are planning to get married in 2021. When I asked him, “Have you been tested for HIV/AIDS? he said,* ‘*I haven't*.’” *And when I asked her,* “*Have you got tested for HIV/AIDS?*” *she said,* “*I haven't.*” *When I asked and said to the guy,* “*Do you assume that you have never been exposed to the risk? he said to me, I extremely fear the virus. I don't even want its name (HIV) to be mentioned.*” *(KII, 40 years old, female*)

##### 3.2.1.3 Attitude on sexual behaviour

According to most study participants' narratives, adolescents should not start Sexual intercourse at an earlier age. They gave different reasons. They explained that there is limited awareness of the risk of contracting STIs after the early initiation of sex. Participants additionally stated that lack of knowledge can lead to risky sexual behaviours that can lead to the contraction of STIs and unplanned teen pregnancy. Furthermore, this may lead to adolescent girls engaging in unsafe abortions. Most adolescents regret having sexual intercourse, as captured in the extracts. Many adolescents have a positive attitude toward the use of condoms during sexual intercourse. They mentioned the importance of condom use for preventing STIs, including HIV/AIDS, unintended pregnancies, and having sex without any fear or frustration. According to health professionals and some adolescents' narratives, adolescents have a limited level of risk perception of multiple sexual partners, though they participate in it. According to the opinion of adolescents, engaging in multiple sexual partners can expose them to different diseases, including HIV and STIs, and ultimately to poor health outcomes. Adolescents have a limitation on risk awareness, and most of them have a negative attitude towards having multiple sexual partners.

“*Adolescents face different problems when they have sex at an early age, like unwanted pregnancy, HIV/AIDS, and STIs. Nowadays, we adolescents like to try everything quickly; adolescents want to experience everything.*” *(IDI, 17 years old, female)*

“*The adolescents are only concerned about unwanted pregnancy. Nobody is concerned about the consequences of having multiple partners. This, I think, is related to a lack of training and knowledge.*” *(KII, 27 years old, male)*

##### 3.2.1.4 Behaviour stigmatization and judgemental

According to the narrative of the participants, adolescent engage in various substance abuse such as alcohol, khat, shisha, and the after the engaged in unsafe and multiple sexual activities. There are conditions such sexual involvement with Sugar mammy and sugar daddy to fitful their financial needs.

“*After some adolescents finished the Ministry exam, they threw a party, and four guys of the same age had sex with one girl; the girl did not know she had sex with four guys, and the boys were friends and were not aware. As I told you, they get high using hashish; they were all compounded children.*” *(KII, 32 years old, female)*.

*There is what we call PESTL; we use this term as an abbreviation for psychological, economic, social, technological, and legalization. These are five things. To fulfil these five things, they (adolescents) want to meet those who are older than them, fulfil their needs, and engage in transgenerational sex. They may have transgenerational sex with what we call sugar mammy or sugar daddy. They have sex with the one who gives them money (KII, 54 years old, male)*.

When adolescents usually need to other place hotel or party for enjoyment, they pretend as if they are going to a birthday party or a ceremony. Participants mentioned that some participants can dress seductively and by so doing, send the wrong message that they are available for sex.

“*Adolescents usually tell their families that they are going to church, school, or the library and then go to other places that they know their parents would not allow. Not knowing that, at the end, it will hurt them.*” *(IDI, 26 years old, male)*

“*Their dressing styles have their influence. For example, if a woman comes to you wearing a nightgown, you may not give her attention, or she may not seduce you. She may not arouse your sexual desire. If you see girls wearing miniskirts that highlight the shape of their bodies, many people will be easily aroused. Therefore, the girls*’ *dress has its influence*.” *(KII, 29 years old, female)*.

#### 3.2.2 Relational level influence

In this theme, different factors such as family, peer, and partner-related factors are included in the following subcategories.

##### 3.2.2.1 A gap in family involvement in sexual and reproductive health behaviour modelling

According to the narratives, child-parent communication about sexual and reproductive health, such as sex and condoms, was perceived as low because talking about it was considered taboo, and some families also lacked the basic skills and knowledge of SRH. It was perceived that families would suspect that their children are already involved in sexual relations if they talk about sex, condoms, or another SRH-related issue. Adolescents with limited family control and supervision are more likely to engage in risky sexual behaviour. Adolescents who live with a grandmother, or relative or other guardian, live alone, or live with only one biological father or mother were more likely to engage in risky sex than those who grew up with both a biological father and biological mother**.**

“*It is very difficult. If you sit and try to talk to your mother about a condom, she will say,*” “*How ill-mannered are you? Stop it.*” *People may say,* “*She must have started sexual relations; that is why she is talking about condoms.*” “*They would question your motive to know the usage of condoms.*” *(IDI, 19 years old, female)*

“*I have a friend who used to live with her grandfather. She gets food and supplies from school. After her grandfather passed away, she became independent. She started spending time in bad places next to our school.*” *(IDI, 17 years old, female)*

The parenting style also matters. Participants in this study also mentioned that parents who are overly strict and controlling make the children rebel, and this leads to adolescents engaging in risky sexual behaviours. On the other hand, failing to control or turning a blind eye has its influence. Therefore, adolescents prefer neither dictatorship nor permissive parenting styles recommending that parenting styles should be democratic with moderate control and open discussion. Besides, if adolescents quarrel with their families, they usually engage in substance abuse, such as drinking alcohol, and finally get involved in risky sexual behaviour.

“*In my opinion, neither being extremely strict nor careless are good. A family should be strict to some extent. It is also important to have some kind of intimacy. A family should guide children properly. Corporal punishment is not good. There should be a discussion with children.*” *(FGD, 18 years old, female)*

“*Family influences you. If you go somewhere alone, when you come back, they suspect that you might have had sex. When they think that way, they insult you. They call you* ‘*Bitch.*’ *These things influence the girl to develop bad behaviour. She wants to try the life that they talk about.*” *(IDI, 18 years old, female)*

##### 3.2.2.2 Peer-related factors

Peer influence is when you choose to do something you wouldn't otherwise do, because you want to feel accepted and valued by your friends. It isn't just or always about doing something against your will. The term “peer pressure” is used a lot. But peer influence is a better way to describe how adolescents’ behaviour is shaped by wanting to feel they belong to a group of friends or peers. Peer pressure and influence can be positive or negative. For example, adolescents might be influenced to become more assertive, try new activities or get more involved with school or might choose to try things they normally wouldn't be interested in, like risky sexual behaviours. Negative peer norms are predominantly more common among males than females because of the permissive attitude toward sexual intercourse for adolescents stated below:

“*Even now, what does he say? … When we were in a training session last year, there was a guy who gave us his opinion by saying,* ‘*Virginity means what you should be ashamed of.*’ *Now, the girls and the boys alike are ashamed to say that* ‘*I am a virgin.*’ *Even if he had no sex at all, he would talk and brag by saying,* ‘*I had sex three times or I had sex with three ladies.*’” *(KII, 27 years old, male)*

“*There is a saying that* ‘*Tell me your friend and I will tell you who you are*’*. If a friend of yours has done it (sex), you feel pressured to do it because you feel inferior to your friend. At this age, we cannot think like a mature person.*’” *(IDI, 19 years old, female)*

Peer-to-peer communication drives adolescents to risky sexual behaviour. The findings of this study also reported that adolescents usually talk about sex, love, and friendship every time and everywhere. They explained that this type of communication repeatedly comes to their mind and try to have a boyfriend or girlfriend when their friend hears about the difference between sex with a condom and sex without a condom, then they want to experience it without a condom. When adolescents talk to each other about sex-related issues, they learn from each other and want to experience what the others have done it.

“*There is one student who talks about different boys who have relationships with her. There are bars and khat shops around her home. She spends time with such people. She told us about a handsome man that she kissed and had sex with her. Due to this, I decided to say ok to the boy who asked me to be his girlfriend.*” *(IDI, 17 years old, female)*

##### 3.2.2.3 Partner and relationship-related factors

Partners are those who have sexual relations with one another. The sexual partners may be in a committed relationship, either on an exclusive basis or not, or engage in the sexual activity on a casual basis. They may be on intimate terms such as lovers or anonymous. These relationships can involve physical, psychological, or sexual abuse, as well as harassment or stalking. Revenge, failing love, lack of trust, maintaining the relationship, conflict with the partner, long- time relationships were commonly the relational problems, and such issues put adolescents in risky sexual behaviour. If some of the male adolescents expect that their girlfriends are cheating with other boyfriends or if they do not care about their girlfriends, they end up having sex without a condom with them (their girlfriends) by considering this as a revenge. Participants also mentioned that when they no longer trust a girl, they will have sex with her and with others. There is a tendency to refuse to use a condom, especially among males. Though there is a lack of awareness about sexually transmitted diseases, female adolescents want to use condoms to prevent unintended pregnancy.

“*When he talks to me, he says that it is* ‘*BATAKOYENG*’ *(having an alternative in partnership). If he misses the first one, he can go to the second one. So, he says that it good thing. You will not be hurt. If you quarrel with the first one, you can hang out with the second one.*” *(IDI, 18 years old, male).*

“*One may want to use condom and the other one may not want to use. This time the male will chose to have sex without condom. Also, if their relationship is real, the males will choose to have sex without condom. But if not, he would rather choose to use condom*”*. (KII, 29 years old, Female.*

“*Sex without a condom is mainly common among guys. The girls want to use a condom more than most of the boys.*” *(KII, 32 years old, female)*

## 4 Discussion

This study explored that various biological factors, limited knowledge, and skill of SRH, Stereotyping and misjudgement, permissive attitude on the early initiation of sex and limited skill, a lack of trust, a few misconceptions about condoms, and substance abuse fuel unplanned sexual relationships. Limited child-parent communication on SRH, parenting style and control, and child and parent conflict have contributed to adolescents' sexual behaviour. On the other hand, negative peer norms, peer pressure, male dominance, sexual violence, and pressuring females to sexual intercourse were found perceived factors influencing adolescents to engage in risky sexual behaviour at the peer and partner level factors.

In this study, adolescents were participating in risky sexual behaviour because of their biological needs. This finding is supported by other findings. For instance, physiological needs related to sexual intercourse drive adolescents to follow their emotions and engage in risky sexual behaviours (45, 46).

TAdolescents living in Addis Ababa were found to have limited knowledge about sexual and reproductive health. They feared pregnancy more than STIs and HIV because of their limited knowledge about the severity of the diseases. Besides, some stereotyping and misjudgement were explained about using condoms. Our study was supported by different studies in the world; lack of knowledge and skill on SHR led adolescents to engage in risky sexual behaviour in Morocco (47). Studies done in Ethiopia revealed that comprehensive knowledge of HIV/AIDS among youth was relatively low, at 35% among males and 24% among females, Youth and adolescents who have a low level of sexual and reproductive health knowledge are more likely to practice unsafe sexual practices because of a lack of information and poor negotiation skills (31). This indicates that the responsible bodies should address the SRH knowledge gap by providing comprehensive sexual education. Because education, especially comprehensive sexual education (CSE), has positive effects including increasing young people's knowledge and improving their attitudes related to sexual and reproductive health and behaviours as they move into adulthood (48).

Almost half of the study participants were found with permissive attitudes toward early sexual initiation, and on the other hand, they claimed that they should not have sexual intercourse predominantly fear of pregnancy and few of them due to fear of diseases such as STIs and HIV AIDS. Study participants were found with a positive attitude toward using a condom during sexual intercourse despite their limited knowledge and skill in using a condom. Perceiving the risks of multiple sexual partnerships was explained by study participants. This study supported that adolescent perceived that premarital sex was risky (46). Study participants believed that condoms could protect them from contracting STIs and pregnancy (47). On the other hand, more than 80% of study participants had a favourable attitude towards premarital sex (49). This implies that despite of adolescents has permissive attitude towards early initiation of sex, they have somehow understandings on risky of acquiring STIs including HIV and unwanted pregnancy secondary to unsafe sex and multiple sexual partners.

Our findings revealed that, adolescents were engaging in risky sexual behaviour because of unplanned sexual relationships and substance abuse. Similar studies indicated that substance abuse was found to be the driver of risky sexual behaviour (50, 51). This might be because the level of mental functions gets lowered by the effects of alcohol. This might hinder them to comprehend things that put them at potential risk to engage in risky behaviours. We would also like to suggest that alcohol consumption among adolescents be better controlled, and that people be made aware of the negative side effects of alcohol and other substances.

On the other hand, dressing style and deceiving behaviour were considered as mediators of risky sexual behaviour because, studies indicated that amorous dressing styles seduce males and drive to engage in sexual violence (52), some women intentionally dress sex-typed but inferences about women who wear sexy dress can be misinterpreted and are sometimes negative (53).

Poor child-parent communication about sexual and reproductive health issues is judged to have a great influence on risky sexual behaviour. Various studies have revealed that child-parent communication about sexual and reproductive health is associated with adolescents' sexual behaviour. Poor child-parent communication due to various cultural factors affects the lack of knowledge and skills of rational decision-making, hindering the seeking contraceptives and negotiating safer sex, resulting in lower self-confidence (54–56). This might indicate that parents can improve the chances of communicating with their children about sex by conveying non-judgemental attitudes, using open communication styles with neutral messages, and appearing comfortable at the same time as displaying positive attitudes towards communication around sex and contraceptive use. There should be mechanism to improve SRH knowledge parent and enhance their communication skill how to approach their children.

We found family control, supervision, and parenting style-related issues were perceived as factors related to risky sexual behaviour. Youth and adolescents who were living alone and without parental control were practicing sex with multiple sexual partners (57). Staying alone increases the reliance of adolescents on peers because there would be no parental monitoring (58). Authoritative parenting was the best parenting styles recommended by most of the study participants in this research. Authoritarian, authoritative parenting styles prevent adolescents from engaging in risky sexual behaviour than other types of parenting style in Addis Ababa (59). When the parenting role is not well handled, there is a tendency for delinquency, and permissive and authoritarian parenting styles significantly predict experimenting with sex, while authoritative parenting styles do not have a significant influence on sex experimentation (60). Authoritative parenting style reduced sexual risk behaviours among adolescents in Ethiopia. So, our findings suggest that the need to address family parenting style focusing authoritative parenting style to enhance healthy SRH behaviours among adolescents (61).

Our study showed that when child-parent conflict occurs, adolescents tend to engage in substance abuse and risky sexual behaviours. It was evident that adolescents who had higher levels of conflict with their parents and lower parental support were predictably at a higher risk of engaging in risky sexual behaviour (62, 63). Result indicated that more rapid increase in mother–adolescent conflict, more rapid decline in father–adolescent closeness, and a more rapid increase in father–adolescent conflict predicted more engagement in sexual behaviours by age 15 (64).

Negative peer norms, peer pressure, and usual peer-to-peer communication about sexually related matters were other important findings associated with risky sexual behaviour. Early initiation of sex and multiple sexual partnerships are considered brave, heroic thinking among peers. Provocative communication leads others to start it. Studies have generally reported in support of our study that adolescents who perceive that their network members approve of sex and sexual practice are more likely to engage in risky sexual behaviour than those who disapprove of sex and sexual practice (65). If participants do not strongly perceive that their peers believe condom use is always a good idea, this perception predicts their intention not to use a condom during sexual intercourse (66). Overall, kinds of literature revealed that peer influence and pressure had an association with risky sexual behaviour (67, 68). A study done in Ethiopia indicated, peer pressure was considering as a major factor that influences secondary and preparatory students towards their first sexual intercourse. Peers had greater influence on the positive and negative behaviour of their friends (69). Therefore, this situation calls up one responsible body should emphasize on promoting peer educators and peer discussion to protect adolescents and youth from risky sexual behaviours.

Failing in love, sexual desire, male dominance of sexual relationships, and pressuring females to sexual activities such as sexual relationships were listed as partner-related factors that predispose adolescents to engage in risky activities. This finding was supported by a study done in Ethiopia showed that students were engaged in risky sexual behaviour with the primary reason of falling in love (70). Nowadays, romantic Relationships are considered as norm despite that their effect are dangers. Adolescents, especially girls who indulge in sexual intercourse before the age of 18 years tend to feel misguided, cheated, and depressed if left by their partner later (71). Therefore, awareness creation programs should be enhanced for adolescents inform them about romantic relationships, the difference between infatuation and love.

According to the narratives of this findings, adolescents are facing problems on safe sexual negotiation skill, shame on buying condom, and some of the female adolescents were enforced to engage sexual violence and unsafe sex dominated by male partners. Similarly, ashamed for asking sexual partner about condom, partner fear of buying condom from shops or pharmacies, lack of interest lack of knowledge to use, and perceived barrier for sexual pleasure were the reasons described for inconsistent or non-condom use among youth and students in Ethiopia (72), It is also conclusive that young girls are pressurized into sexual activities (73), and sexual violence (74), Almost 45% of the study participants had experienced sexual violence in their lifetime, those 15. 5% of them had ever been exposed to rape (75), by neighbours, family members, friends, employers, teachers, and strangers (76). Sexual abuse among males is also a problem in Ethiopia (77).

### 4.1 Research contribution

TThe study found that biological, Limited knowledge and skill on SRH, dressing style, poor economical statues, substance abuse, permissive attitude on early initiation of sex where factors are driving adolescents to engaged in risky sexual behaviour. The findings also reveal permissive and authoritarian Parenting style, child-parent conflict, negative peer pressure and peer norm, male dominance of sexual relationship and sexual violence influence adolescent to engage in risky sexual behaviour. The study provides a new insight, female adolescents fear to pregnancy than HIV because they have lower knowledge and low perceived severity of HIV/AIDS. These findings are original and contribute to the literature on the effect of individual and relational level factors on risky sexual behaviour, providing insights that can inform policies and practices for improving sexual behaviour of adolescents.

## 5 Conclusion

Risky sexual behaviour in this study was influenced by various individual and relational-level factors. Generally, the perceived factors that influence adolescents to engage in risky behaviour reported in other studies are like what we found in the perceived factors that influence adolescents to engage in risky behaviour in this study. However, this finding indicated new insight: the gaps in SHR knowledge among adolescents, especially on HIV/AIDS and STIs, influence female adolescents to fear pregnancy more than STIs, including HIV/AIDS. Responsible bodies should introduce socially and culturally acceptable, comprehensive sexual education curricula for in-school and out-school adolescents to enhance SRH knowledge, attitude, and skill. There should be SRH strategies or interventions that will benefit adolescents when interacting with parents, peers, and partners.

## 6 Strength and limitation

The limitation of this paper is that the topic was a very sensitive one, requiring adolescents to verbalize their experiences and share their thoughts with the researcher. The age of the researcher might have been a limitation too. The study was conducted in one area among homogenous people who have the same experiences and challenges. A richer database could have been achieved if the study was done in two or three other different regions of the country.

In addition, adolescent sexual behaviours are highly sensitive topics; consequently, talks could have resulted in participants withholding crucial information from the researcher. These constraints tried to be overcome by informing participants that their Candor would be crucial for recommending targeted interventions to prevent adolescent from risky sexual behaviour by assuring their anonymity.

The study was conducted in a familiar place for participants. This created an environment with a friendly atmosphere for developing relationships with one another. The researchers remain sensitive to their own assumptions about adolescents’ engagement in risky sexual behaviours to eliminate the researcher's bias. We applied various strategies, such as building rapport, self-disclosure, reciprocity, and appropriate and sensitive use of open questions. In additions to this we were ensuring a comfortable environment and appropriate timing. These measures helped us at to gain trust from our participant and enhance spontaneous exchange of information in a warm and supportive environment. Because talking about an experience in a safe and respectful environment can help with gaining closure and personal control or efficacy over the event or situation.

## Data Availability

The raw data supporting the conclusions of this article will be made available by the authors, without undue reservation.
