# Supplementary material for: Individual and relational dynamics perceived to influence the sexual behaviour of adolescents in Ethiopia: a qualitative study
Source: Front Reprod Health. 2024 Aug 6;6:1348953. doi: 10.3389/frph.2024.1348953 (PMC11333451; doi:10.3389/frph.2024.1348953)
Supplement: Supplementary file 1 [file Datasheet1.docx]

**S1. Consolidated criteria for reporting qualitative studies (COREQ): 32-item checklist**

| **No. Item** | **Guide questions/description** | **Reported on Page #** |
| --- | --- | --- |
| **Domain 1: Research team and reﬂexivity** |  |  |
| *Personal Characteristics* |  |  |
| 1. Interviewer/facilitator | **Which author/s conducted the interview or focus group?**  Data collection was carried out by the principal investigator (SGB) and two experienced female research assistants. The two female research assistants were assigned to take notes during the FGDs after a brief training about the research ethics and process by SGB | Methods, page 7 |
| 2. Credentials | **What were the researcher’s credentials? E.g. Ph.D., MD**  The authors’ credentials are as follows:   - Semere G baraki., MSc, PhD-candiate - Gloria B Thypaungale PhD, Professor | N/A |
| 3. Occupation | **What was their occupation at the time of the study?**  SGB: Doctoral Candidate  GBT: Supervisor, Professor | N/A |
| 4. Gender | **Was the researcher male or female?**  The authors’ identified genders are as follows:  SGB: Male  GBT: female | N/A |
| 5. Experience and training | **What experience or training did the researcher have?**  -SGB: quantitative and qualitative (advanced and basic) training and extensive experience delivering courses related to maternal and child health, Adolescensts, including sexual and reproductive health program, monitoring and Evaluation, data management and information use, Abortion , Urban health extension , HIV/AIDS, Familly planing  GBT: quantitative and qualitative training and extensive experience in global maternal and child health, including sexual and reproductive health care adolesceents, senior researcher on adolescent health | N/A |
| *Relationship with participants* |  |  |
| 6. Relationship established | **Was a relationship established prior to study commencement?**  SGB has more than 15 years in community health program management in Ethiopia and worked in different contexts of health programs implementation such as unrban health exentesion program , community health service and research evaluation in Ethiopia, had established communication with Sr fetilework haile, who is the SRH of adolescent and youth director in Addis Ababa city Adiministartion health Bureau | N/A |
| 7. Participant knowledge of the interviewer | **What did the participants know about the researcher? e.g. personal goals, reasons for doing the research.**  He explained the purpose and procedure of study and asked them for voluntary participation. Consent form was secured from health professionals before conducting in-depth interviews. | Methods, page 7 |
| 8. Interviewer characteristics | **What characteristics were reported about the interviewer/facilitator? e.g. Bias, assumptions, reasons and interests in the research topic**  Two female research assistants were assigned to take notes during the FGDs after a brief training about the research ethics and process by SGB. The research assistants were fluent in local Amharic language(FGD participants’ working language), one has MSc degree and and and the other has PhD in public health and has experience in qaultative research . | Methods, page 7 |
| **Domain 2: study design** |  |  |
| *Theoretical framework* |  |  |
| 9. Methodological orientation and Theory | **What methodological orientation was stated to underpin the study? e.g. grounded theory, discourse analysis, ethnography, phenomenology, content analysis**  A qualitative Phenomenological study design was conducted with IDIs, FGDs and KIIs to achieve the objectives of the study. This design enables the investigator to explore the phenomena from the perspective of the participant being studied (35). We approached the research questions from the perspectives of health care provider and adolescents about sexual behaviours of adolescents based on individual level, and relational factors | Methods, page 5 |
| *Participant selection* |  |  |
| 10. Sampling | **How were participants selected? e.g. purposive, convenience, consecutive, snowball**  The researchers used purposeful sampling techniques till saturation had been reached | Methods, page 5-6 |
| 11. Method of approach | **IDIs recuritement**  The principal investigator (SGB) worked with the Addis Ababa regional health bureau adolescents and youth case team leader, and the head of selected health care organizations and youth centres. First the principal investigator collaboration with head of selected health care organizations and youth centers purposefully selected various service areas such as Voluntary counselling and testing (VCT), Abortion service units, family planning, Anti-retroviral (ART) clinics, adolescents, and youth outpatient departments. Health professionals working in the selected department and were requested to identify adolescents who met the inclusion criteria. The researcher informs adolescents about the purpose, objective, risk, and benefits of the research. The researcher provided “yes” or “no” question on previous history of sexual intercourse to fill individually. Adolescent who said “yes” for previous history of sexual intercourse asked their willingness for participating in the research process in the IDIs.  **FGDs recuritement**  The principal investigator and the head of youth center selected different service areas, recreation area, clubs, and libraries the selected youth center and communicated with health professionals to call adolescents for orientation about the research. After giving briefing, the researcher himself identified those who fulfilled the criteria purposefully. In both FGDs and IDIs, Assent form was given to adolescents who were willing to participate in the study and Consent form was given to each adolescent for his/her parents to review and determine whether to allow their children to participate in the study the adolescents were found less than 18 years old. The data collection was conducted immediately after the biographic data filled If the adolescents were above 18 years old. But The researcher appointed the participants for the convenient time and place for the next time for adolescents who were less than 18 years.  **KIIs recuritement**  For the sake of KIIs, the principal investigator met the head of health institution and asked to recommend senior health professionals who have experience with adolescents sexual and reproductive health. He explained the purpose and procedure of study, asked them for voluntary participation. Consent form was secured from health before conducting in-depth interviews. | Methods, page 6-7 |
| 12. Sample size | **How many participants were in the study?**  The data was collected from 51 individuals (8 health professionals and 43 adolescents) | Methods, page 5 |
| 13. Non-participation | **How many people refused to participate or dropped out? Reasons**?  None | N/A |
| *Setting* |  |  |
| 14. Setting of data collection | **Where was the data collected? e.g. home, clinic, workplace**  . Data collection took place at different locations depending on participants. The FGDs were conducted in youth centers, IDIs were collected in the youth centesr and Health care organization, while the KIIs were conducted in their offices, and the clinics | Methods, Page 10 |
| 15. Presence of non-participants | **Was anyone else present besides the participants and researchers?**  No, non-participants were present during the group discussions. | N/A |
| 16. Description of sample | The researcher collected data from adolescents, and health professionals; 12 IDIs, 8 KIIs, and five FGDs. Out of the five FGDs, three were conducted with male participants, while two were conducted with female participants. Sociodemographic characteristics such as age, sex, religion, grade, living condition, history of sexual exposure, living situation, and the sub-city were collected during the data collection. The adolescents were represented as P1, P2, P3, etc. Codes during the interview. KII represents the key informant interview, IDI represents the individual in-depth interview, and FGD represents focus group discussion. A total of 43 adolescents participated in the study; out of this, 30 were school attendants, and 13 were out-of-school adolescents  The socio-demographic characteristics of health professionals who participated in the study were also presented. The average age of the study participants in this group was 35.75 years. The oldest was 54 years old, and the youngest was 24 years old. They had an average of 13.50 years of work experience, with a maximum of 32 and a minimum of 4 years of work experience. There were 4 females and 4 males. Regarding their profession, four of the totals were clinical nurses, two were nurse counsellors, and the rest were health officers. All participants were working in a health canter, hospital, NGO, or youth centre. | Result  10-11 |
| *Data collection* |  |  |
| 17. Interview guide | **Were questions, prompts, guides provided by the authors? Was it pilot tested?**  SGB and GBT carefully crafted the interview guides in English and SGB translated to Amharic languages. The interviewed guid was pretested before actual data collection by two adolescents and two health professionals who were not included in this study. | Methods, page 6 |
| 18. Repeat interviews | **Were repeat inter views carried out? If yes, how many?**  Repeat interviews were not carried out. | N/A |
| 19. Audio/visual recording | **Did the research use audio or visual recording to collect the data?**  IDIs, FGDs and KII were all audio-recorded after obtaining participants’ permission to record. | Methods, page 8 |
| 20. Field notes | **Were ﬁeld notes made during and/or after the interview or focus group?**  Yes, The two female research assistants were assigned to take notes during the FGDs after a brief training about the research ethics and process by SGB | Methods, page |
| 21. Duration | **What was the duration of the inter views or focus group?**  The data collection process lasted on average 40–75, 30-75, and 75-115 minutes for IDIs, KIIs, and FGDs, respectively. | Methods, page 9 |
| 22. Data saturation | **Was data saturation discussed?**  In qualitative research, participants are selected due to lived experience with the area of interest, purposeful sampling, to get rich and thick data about the phenomenon of interest and data collection continued until data saturation had been reached or no new concept is emerging (37). The researchers used purposeful sampling techniques till saturation had been reached. In a largely deductive approach, saturation may refer to the extent to which predetermined codes or themes are adequately represented in the data whereas inductive approach thematic saturation in related to the non-emergence of new codes or theme (38). So, the sample size was determined by information redundancy or saturation level, which occurred when no new information, theme, or coding emerged from the data. To ensure saturation, the data was reviewed at the end of each interview day for the presence of codes or categories, as well as the necessity for further interviews in a preliminary manner  . | Methods, page 5-6 |
| 23. Transcripts returned | **Were transcripts returned to participants for comment and/or correction?**  To maintain dependability, four Study participants ( two health professionals and two adolescents) were given raw transcripts by the researchers and inquired whether the transcripts accurately reflected the conversations that took place during the data collection phase | trustworthnes  page 9 |
| **Domain 3: analysis and ﬁndings** |  |  |
| *Data analysis* |  |  |
| 24. Number of data coders | **How many danta coders coded the data?**  SGB coded the data and GBT comment and approve the coding. | Methods, page 8 |
| 25. Description of the coding tree | **Did authors provide a description of the coding tree?**  After reviewing four transcripts and field notes, he developed a code tree with a list of deductive and inductive codes from the data. The deductive codes came from topics in the interview guide and inductive codes captured new themes that emerged in the data (42). | Methods, page 8 |
| 26. Derivation of themes | **Were themes identiﬁed in advance or derived from the data?**  SGB developed a codebook, and this codebook was reviewed by the research supervisor GBT, and revisions were made where necessary till the end of data analysis. Identified discrepancies were discussed, resolved, and reviewed further until consensus was reached. The researchers systematically grouped the sub-categories into categories, and categories into general themes. We organized the findings by major themes, and we discussed minor themes in the manuscript and situated them within the broader literature. Participants’ quotations were presented to illustrate themes and findings. | Methods, page 8 |
| 27. Software | **What software, if applicable, was used to manage the data?**  All 25 transcripts were analysed with ATLAS.ti version 7 software | Method  page 8 |
| 28. Participant checking | **Did participants provide feedback on the ﬁndings?**  No, the participants did not provide feedback on the findings | N/A |
| *Reporting* |  |  |
| 29. Quotations presented | **Were participant quotations presented to illustrate the themes/ﬁndings? Was each quotation identiﬁed? e.g. participant number**  Participants’ quotations were presented to illustrate themes and findings. | Results, pages 9 |
| 30. Data and ﬁndings consistent | **Was there consistency between the data presented and the ﬁndings?**  Yes. | Results, pages 10-21 |
| 31. Clarity of major themes | **Were major themes clearly presented in the ﬁndings?**  Yes, we organized the findings by major themes. | Results, pages 12-21 |
| 32. Clarity of minor themes | **Is there a description of diverse cases or discussion of minor themes?**    Yes, we discussed minor themes in the manuscript and situated them within the broader literature. | Discussion, pages 14-25 |
