# Supplementary material for: Individual and relational dynamics perceived to influence the sexual behaviour of adolescents in Ethiopia: a qualitative study
Source: Front Reprod Health. 2024 Aug 6;6:1348953. doi: 10.3389/frph.2024.1348953 (PMC11333451; doi:10.3389/frph.2024.1348953)
Supplement: Supplementary file 2 [file Datasheet2.docx]

# Supportive file 2: Interview Guide

# PART 1: SEMI-STRUCTURED INTERVIEW GUIDE FOR ADOLESCENTS

Code----------------------

**Part A: Biographic data**

1.1. Age----------------------------

1.2. Sex---------------------------------------

1.3. Religion-----------------------------------

1.4. Educational status/grade----------------------------

1.5. Ethnicity------------------------------------------------

1.6. Average Personal income------------------------------- birr

I do not have any personal income.

1.7. Average Household/ parental income -------Birr

we do not have any parents’ income.

- 1. Employment:

1. Student
2. Government employees
3. Private business
4. Unemployed
5. Others. specify-------------------------------------------------
   1. . Living situation
6. Living alone
7. Living with parents (mother and father)
8. Living with my mother only
9. Living with my father only
10. Living with other family
11. Living with Legal guardian

|  | 1. Living with partner 2. VI. Living in college or dormitory   1.10. Do Have you any of these behaviour s? (you can select more than one behaviour s if you had have it in practice)   1. Smoking cigarette 2. Alcohol drink 3. Chat chewing. 4. Shisha 5. Drug/ medication/tablet or injection abuse. 6. No I do not have any of the above behaviour s   1.11 school attendance at currently?   1. In school 2. out of school   1.12 sub-city of the study participant  1.13 Have you ever had sex?   1. Yes 2. No   1.14. If your answer is yes for question 1.13, What was your age when you first had sex?  1.15. If your answer is yes for question number 1.13. How money lifetime sexual partner have you had?  1.16. If your answer for question 1.13 is yes, do you used condom for sexually transmitted infections and pregnancy during sexual intercourse? **(for** those who are sexually active only).   1. Sometimes use it 2. Usually use it 3. Always I use it 4. I have never used it   1.17. Reason for the last visit to health care organization or youth centre visit?  **Part B: Perceptions on sexual intercourse during adolescence**   \|  \| 1. Do adolescents here have relationship such as girlfriend or boyfriend? 2. What does it mean for adolescents to have a boyfriend or a girlfriend? 3. What are your thoughts about initiating sexual intercourse during adolescence? \| \| --- \| --- \| \|  \| **Probing**: -   - - What in your opinion is good about having sexual intercourse during adolescence? Please explain.   - What in your opinion is bad about having sexual intercourse during adolescence? Please explain.   - Do you see sexual intercourse as pleasurable? Please explain it (when, in what circumstances?).   - What would you advise adolescents should do when decided to have sexual intercourse?   4. Tell me what you think other people’s views are about adolescents initiating0f sexual intercourse?   - Do you think parents and other members of the community allow adolescents (pear)to have sexual intercourse? Please explain. - Tell me what you think the views of teachers are about adolescents having sexual intercourse? Please explain. - Tell me what you think other adolescents think of adolescents who have sexual intercourse? Please explain. - Tell me what you think the religious institution’s view are on adolescent having sexual intercourse? Please explain. - Do you know what the culture of Ethiopia say about adolescent having sexual intercourse? Gender difference, early marriage, early childbearing   5. Tell me Who influences adolescents to have or not have sex intercourse?  6. Tell me what influences adolescents having or not having sex intercourse?   - Tell me how you see the role dressing style in influencing adolescent to initiate early sex practice? How and why? - Tell me how you see the influence of community/ families in adolescent to initiate early sex practice? How and why? - Tell me how you see the role of alcohol in adolescent’s sex practice? How and why? - Tell me what you think the role of media (TV, radio, social media, internet, film, music) is on adolescents to for initiation of sexual intercourse? - Tell me what you think the use of substances on adolescent for initiation of sexual intercourse? - What do you think are school environment influence on adolescents to have or not to have sexual intercourse. - What are the incidences than can drive adolescents lead them to sexual intercourse at early age? - Do adolescents get/give any gift for having sex? Do you think that gift influenced their behaviour? who give any gifts?   **PartC: Sexual experiences**  7.Now let’s discuses from your / friends sexual experience, Could you please describe your first experience of penetrative sexual intercourse?   - - At what age do did you start having sex (sexual Debut) ?   - Tell me where you met your first sexual intercourse partner?   - How long had you known him / her before you had sexual intercourse?   - Did anyone influence you to have penetrative sexual intercourse/ Please explain?   - What was your motivation for sex? Could you please tell me the reasons for engaging in first intercourse?   - Did you feel pressurized into having first sex? By whom?   - Were you given any gifts? Do you think that influenced your behaviour?   - Did you give any gifts? Do you think that influenced your partners' behaviour? \| \|  \| **Part D: Condom use**   1. Tell me what you think would cause some problems for adolescents who have sexual intercourse? 2. Probe, HIV, STI, unintended pregnancy. What are the ways of preventing HIV, STI and pregnancy? Probe into abstinence, modern and traditional contraceptive methods   9. What do you think of using condom during sex? Why are some using whereas others are not using it? Tell me the advantage and disadvantage of condom use?  10. Tell me who can influence adolescent to practice sexual intercourse with out condoms?   - What are the incidences (time, person, relationship) adolescents can practice sexual intercourse without condom? - Tel me about the accessibility of condom (where you found, cost, social norm) for adolescents. - What you perception on the Knowledge, skill, and confidence of adolescent about condom use. - Whose responsibility is condom use during sexual intercourse? Male or female? both? Why? - How do adolescents decide when and when not to use them? - Do you think it is easy to discuss the issue of condom with their parents among adolescents? In what instances has it been difficult? - What are the perceptions of some of the peoples around you (family, friend, health professionals, school perspective and the community you live) about adolescents who carry, buy, and use condoms?  1. Let’s discuses about you/your fried experience: have you you/ your friends experience sexual intercourse without condom? What was the reason doing these activities.   **Part E: Multiple Partners and social influences**   1. Tell me your opinion about having more than one sexual partner?  - What will be the benefits, risky of having more than one sexual partner? - Tell me what you think the views family adolescents have multiple sexual partners?  1. What do you think are reasons why adolescents have more than one sexual partner?  - Do you think the community you live influence adolescents to practice sexual intercourse with multiple sexual partners? Please explain it ? - Do you think peers influence to practice sexual intercourse with multiple sexual partners? Please explain how could it influence? - Do you think school environment influence adolescents to practice sexual intercourse with multiple sexual partners? Please explain how could it influence? - Do you think the need for money influences adolescent to have more than one multiple sexual partners? Please explain. - Do you think material gains such as gifts influence adolescent to have more than one sexual partner? Please explain.   - Do you have any friends who have multiple sexual partners?   - What do your friends say on having more than one sexual partner?   **Summery questions**   1. As you have told me in the previous discussion, there is early initiation of sex among adolescents, there is inconsistent condom use among adolescents, a and there is multiple sexual partner practice, so what should be done to prevent all this risky sexual behaviour practices among adolescents, and whose responsibility is this? 2. Can you add anything that is left un addressed issue that should be raised that you thing as relevant?   **Thank you for your response!** \| \|  PART 2: FOCUSED GROUPINTERVIEW WITH ADOLESCENTS Code: ----------------------  PART A: Biographic data  1.1. Age----------------------------  1.2. Sex---------------------------------------  1.3. Religion-----------------------------------  1.4. Educational status/grade----------------------------  1.5. Ethnicity------------------------------------------------  1.6. Average Personal income------------------------------- birr  I do not have personal income.  1.7Do you know you family income   - 1. yes   2. no   1.8. Average Household/ parental income -------------------------Birr  my parent do not have any income  1.9. Employment:  I. Student   1. Government employees 2. Private business 3. Unemployed 4. Others. specify-------------------------------------------------    1. . Living situation of the adolescents 5. Living alone 6. Living with parents (mother and father) 7. Living with my mother only 8. Living with my father only 9. Living with other family 10. Living with Legal guardian 11. Living with partner 12. Living in college or dormitory   1.11. Do Have you any of these behaviour s? (you can select more than one behaviour s if you had have it in practice)   1. Smoking cigarette 2. Alcohol drink 3. Chat chewing 4. Shisha 5. Drug/ medication/tablet or injection abuse 6. No I do not have any of the above behaviours   1.12. Current school attendance  I. In school  II.Out of school  1.13 . Sub city of the study participants ----------------------------------------  1.14. Have you ever had sex ?   1. Yes 2. No   1.15. If tour answer is yes for question 1.14, What was you age when you first had sex ?  1.15. If yes for question number 1.14 is yes, how money lifetime sexual partner have you had? -----------------------------------------  1.16. If your answer for question 1.14 is yes, Do you used condom for sexually transmitted infections and pregnancy during sexual intercourse? ( for those who are sexually active only)   1. Sometimes use it 2. Usually use it 3. Always I use it 4. I have never used it   1.17 Reason for the last visit to health care organization or youth centre visit --------------   \|  \| **Part B: Perceptions on sexual intercourse during adolescence**   1. What does adolescent age mean to you? 2. What are the problems faced by adolescents related to sexuality? \| \| --- \| --- \| \|  \| 1. Let’s as discuses about sex? What is your opinion about sexual intercourse at the age o you are in? Does your friends share the same view as yourself about early initiation of sex or have different ideas ? 2. What about the community you live?   **Probe፡-**   - What do your family think on adolescents on having or have not sexual intercourse at early age? Why? - What are school perspectives perceptions on adolescents have or have not sexual intercourse at early age? Why? - Let us now talk about perspectives of religious organization and leaders in adolescents having or not having sexual intercourse at early age? - What do you think will be the perception of the community towards sexual intercourse at the age you are in?. - In your Culture is sexual intercourse by adolescents acceptable or not? Why  1. What do you think the advantage and disadvantage of sex at the age you are in? 2. At what age do you think are most adolescents are usually practicing sexual intercourse & who influence them to start it? 3. Tell me what are the driving factors influences adolescents to have sexual intercourse?   **Probe ፡**   - what do you think is the role of social media on have sexual intercourse at adolescent age?   what do your think is the role occulture sexual intercourse of adolescents?   - what do your think is the role of poverty or level of income to have sexual intercourse? - Do you think alcohol influence or does not influence sexual intercourse? How and why? - How about using substance such as chat, drugs, or shisha? How and why? - Do adolescents get any gift for having sex? Do you think that influenced their behaviour? who give any gifts? Do you think that can influence sexual partners' behaviour?   **PART C: SEXUAL EXPERIENCE**   1. Now lets discuses from the experiences of your friends, Can you describe their first experience of penetrative sexual intercourse?  - Tell me where their first sexual intercourse partner meets each other? - How long had they known each other before they had sexual intercourse? \| \|  \| **Part D: condom use** \| \|  \| 1. Tell me what you think would cause some problems for adolescents who have sexual intercourse?   **Probe**, HIV, STI, unintended pregnancy. How do they usually prevent those problems?  10.What do you think of using condom during sex? Why are some using whereas others are not using it?  11.. Tell me the advantage and disadvantage of condom use?  12. What are the perceptions of some of the peoples around you (family, friend  health professionals, school perspective and the community you live) about adolescents who carry and buy condoms.  13. Tell me who can influence adolescent to practice sexual intercourse with out condoms?   - What are the incidences (time, person, relationship) adolescents can practice sexual intercourse without condom? - Tel me about the accessibility of condom (where you found, cost, social norm) for adolescents. Could this be a reason for not using condom? - . What you perception on the Knowledge, skill, and confidence of adolescent about condom use. - Whose responsibility is condom use? Male or female, both? Why? - How do adolescents decide when and when not to use them? - Do you think it is easy to discuss the issue of condom with their partners among adolescents? In what instances has it been difficult?   14. Lets discuses from the experience of your friends or what you hear: if friends experience sexual intercourse without condom? What was the reason doing these activities.  **PART E: Multiple partners and socio-cultural influence**  15. Tell me your views about having more than one sexual partner?   - Tell me what you think the views of your family are towards multiple sexual partners? - Does your any culture in the community you livesupport multiple sexual partners practice? Please explain - . What will be the benefits and benefit having more than one sexual partner?  1. What do you think are the reasons why some adolescents have more than one sexual partner?  - Do you think social environment (families, peers, community, school, religious community) influence adolescents to practice sexual intercourse with multiple sexual partners? - Do you think the need for money /material influences adolescent to have more than one multiple sexual partners? Please explain.   **Summery questions**   1. As you have told me in the previous discussion, the is early initiating of sex among adolescents, there is inconsistent condom use among adolescents, and there is multiple sexual partner practice, so what should be done to avoid all this risky sexual behaviour practices among adolescents, and whose responsibility is this. 2. Can you add anything that is left unaddressed issue that should be raised that you thing as relevant?   **Thank you for your response.** \|  PART 3: KEY INFORMANTI NTERVIEW WITH HEALTH PROFESSIONALS Code: ---------------------------  **PART A: Biographic Data**   1. Age------------------------ 2. Sex---------------------------- 3. Profession---------------------------- 4. Educational status --------------------- 5. Marital status------------------------ 6. Work experience in year---------------- 7. Sub city -------------------------------------- 8. Religion------------------------------------ 9. Ethnicity -------------------------------------  \|  \| \| 1. What does mean adolescent s age to you? 2. What are the current health problems among adolescents in the community where you live? what about In your health institutions? \| \| --- \| --- \| --- \| \|  \| 1. In your opinion, do you think adolescents should start sexual relationship? Why? What about by the community?   **Probing:-**   - What is the perception of the families about initiating of sexual intercourse during adolescent’s age? - What is the perception of the adolescent them self about initiating of sexual intercourse during adolescent’s age? - What is the perception of the school environments about initiating of sexual intercourse during adolescent’s age? - Is it culturally acceptable to start sexual intercourse during adolescent age or not   4.What do you think on the magnitude of initiating of sexual intercourse among adolescents? Lets talk on the age majority of adolescents start sexual intercourse from you experience in the service you provide?  5. Tell me who influences adolescents to have sex intercourse?  6. From you work experience and opinion, could you explain what influences/ drive adolescents to initiate sex intercourse.  **Probe:**   - what do you think social media play any role to initiate sexual intercourse among adolescents? - what do you think alcohol play any role to initiate sexual intercourse among adolescents? - what do you think substance such as chat, drugs, or shish play any role to initiate sexual intercourse among adolescents? How and why? - What do you think is the role of friends and peers on adolescents to initiating sexual intercourse at early age? - What do you think is the role school environment on adolescents to initiating sexual intercourse at early age? - Do you think money/material gain will influence adolescents initiating sexual intercourse at early age?   What do you think is the role social relationship (marriage, party, and birthday) on adolescents to initiating sexual intercourse at early age?  7. How do you evaluate the level of knowledge among adolescents on health outcomes of sexual intercourse during adolescent age?  8. Tell me what are the problems facing adolescents from the service you provide?  **Probing: -**   - HIV, STI, unintended pregnancy? - Do you think that adolescents are properly preventing or not preventing the problems listed above. How? Why?   9. From your work experience, what are the reasons/ factors that affect adolescent to practice sexual intercourse with out condoms?  **Probe: -**   - What are the incidences adolescent can practice sexual intercourse without condom? - Are there any myths/wrong perceptions about condom among adolescents? please tell me. - What do you think about the Knowledge of and skill of adolescent about condom use.   - What do you think the challenges on accessibility of condom for adolescent?   Interns of cost, buying, carrying, confidence,   - - How do adolescents decide when and when not to use condom?   Have you seen difference on using condom in terms of age, sex, religion, education? Please explain it,  10.How do you explain the health service to adolescents in your institution?  11. As health professionals what do you teach adolescents regarding condom use?  **PART E: MULTIPLE PARTNERS AND SOCIO-CULTURAL INFLUENCE**  12. How prevalent is multiple sexual partners among adolescents?  13. What factors do you think adolescents drive them to practice sexual intercourse with multiple sexual partners?  14. Are there some social and cultural factors that led adolescents to practice sexual intercourse with multiple sexual partners?  15. How do you think social activities (social media) influence adolescents to practice sexual intercourse with multiple individual?  16. From the community your live (family, pear, environment, school, religious environment) do you think they influence adolescents to practice sex with multiple sexual partners? Please explain it.  17. Do you think money/material gain will influence adolescents practice sex with multiple sexual partners? Please explain it.  18. What is knowledge adolescents regarding disadvantaged of multiple sexual partners.  **Summery questions**  19. As you have told me in the previous discussion, the is early initiation of sex among adolescents, there is inconsistent condom use among adolescents, and there is multiple sexual partner practice, so what should be done to preventable those risky sexual behaviour practices among adolescents, and whose responsibility is this?   1. Can you add anything that is left unaddressed issue that should be raised that you thing as relevant?   **Thank you for your response.** \| \| \|  \| \|  \| \|  \| \|  \| |
| --- | --- | --- | --- | --- | --- | --- | --- | --- | --- | --- | --- | --- | --- | --- | --- | --- | --- | --- | --- | --- | --- | --- | --- | --- | --- | --- | --- |
